# Heparinized chitosan stabilizes the bioactivity of BMP-2 and potentiates the osteogenic efficacy of demineralized bone matrix

**DOI:** 10.1186/s13036-020-0231-y

**Published:** 2020-03-06

**Authors:** Soyon Kim, Jiabing Fan, Chung-Sung Lee, Chen Chen, Ksenia Bubukina, Min Lee

**Affiliations:** 1 Division of Advanced Prosthodontics, University of California, Los Angeles, USA; 2 Department of Bioengineering, University of California, Los Angeles, USA

**Keywords:** Heparin, Hydrogel, Bone morphogenetic protein, Demineralized bone matrix, Osteogenesis

## Abstract

**Background:**

Demineralized bone matrix (DBM), an allograft bone processed to better expose osteoinductive factors such as bone morphogenetic proteins (BMPs), is increasingly used for clinical bone repair. However, more extensive use of DBM is limited by its unpredictable osteoinductivity and low bone formation capacity. Commercial DBM products often employ polymeric carriers to enhance handling properties but such carriers generally do not possess bioactive functions. Heparin is a highly sulfated polysaccharide and is shown to form a stable complex with growth factors to enhance their bioactivities. In this study, a new heparinized synthetic carrier for DBM is developed based on photocrosslinking of methacrylated glycol chitosan and heparin conjugation.

**Results:**

Heparinized chitosan exerts protective effects on BMP bioactivity against physiological stressors related to bone fracture healing. It also enhances the potency of BMPs by inhibiting the activity of BMP antagonist, noggin. Moreover, heparinized chitosan is effective to deliver bone marrow stromal cells and DBM for enhanced osteogenesis by sequestering and localizing the cell-produced or DBM-released BMPs.

**Conclusions:**

This research suggests an essential approach of developing a new hydrogel carrier to stabilize the bioactivity of BMPs and improve the clinical efficacy of current bone graft therapeutics for accelerated bone repair.

## Background

Autologous bone grafts have been considered the “gold standard” for bone regeneration. However, bone grafts are limited by donor sites morbidity, extended operative time, and autogenous supply [1]. Demineralized bone matrix (DBM) is processed to reduce immunogenicity and has been used widely in the orthopedic industry [2, 3]. During processing, the mineral content of DBM is removed to better expose the osteoinductive factors present in DBM particularly bone morphogenetic proteins (BMPs), powerful regulators for bone formation [4]. However, the processing also eliminates stem cells and the osteoinductivity of DBM can be variable, and clinical failure in orthopedic applications is reported high compared to autograft bone [5]. Moreover, the released osteoinductive cytokines will easily lose their biological function due to their inherent proteolytic and chemical hydrolysis in vivo [6]. Commercially available DBM products often use liquid carriers but they have poor irrigation resistance following implantation, diminishing a localized effect of osteogenic components at the bone defects [7]. Exogenous recombinant BMPs can be employed to improve osteogenic effects of DBM [8]. However, their clinical applications require a supraphysiological concentrations potentially leading to various side effects [9] such as undesired ectopic bone development [10], cyst formation [11], osteoclastic resorption [12], or adipogenesis [13]. Therefore, there is a need to develop an alternative biological carrier that can enhance the potency of BMPs present in DBM and better guide the osteogenic response at the defect site.

Heparin is a highly sulfated polysaccharide in extracellular matrix and has been widely studied as a delivery carrier of growth factor proteins including BMPs [14–16] due to its high binding affinity to the proteins. It has also been shown to maintain or enhance the biological activities of growth factors by forming a stable complex [17]. Heparin can act as a catalyst to form the signaling complexes of BMPs by recruiting type II receptor subunits, even though it has no direct activation role [18]. Heparin is also known to regulate BMP functions by interacting with BMP antagonists such as noggin. Heparin has been shown to regulate the diffusion of noggin in vivo and form the gradients of BMP-2 activity by inhibiting its binding to BMP [19–21].

Chitosan is a biocompatible and biodegradable polysaccharide [22] that has been well studied in bone tissue engineering applications [23, 24]. Chitosan can be easily modified with different functional groups and readily forms three-dimensional networks by simple processing such as thermogelling [25] or photocrosslinking [26]. Recent studies report the use of chitosan as composites with other polymers or ceramics for improved mechanical properties [27].

In this work, we report a novel heparinized synthetic carrier for DBM that can stabilize and enhance the bioactivity of BMPs for increased osteoinduction. We previously reported a light-inducible hydrogel system composed of methacrylated glycol chitosan (MeGC) as a photocrosslinkable polymer and riboflavin as a photoiniator [26]. The MeGC system is highly biocompatible to support the growth of encapsulated cells and can be readily modified with different functionalities such as cell-adhesive-motifs [23] or matrix-degradable-unit [28]. Herein, we immobilized heparin to MeGC by simple chemical conjugation and investigated whether the heparinized MeGC can have protective effects on BMP-2 for osteogenesis under physiological stressors found under bone fracture healing or in the presence of BMP antagonist, noggin. We also demonstrated the potential of heparinized MeGC as biological carriers for DBM to enhance osteogenic efficacy.

## Results

### Characterization of heparinized chitosan

**Fig. 1:**
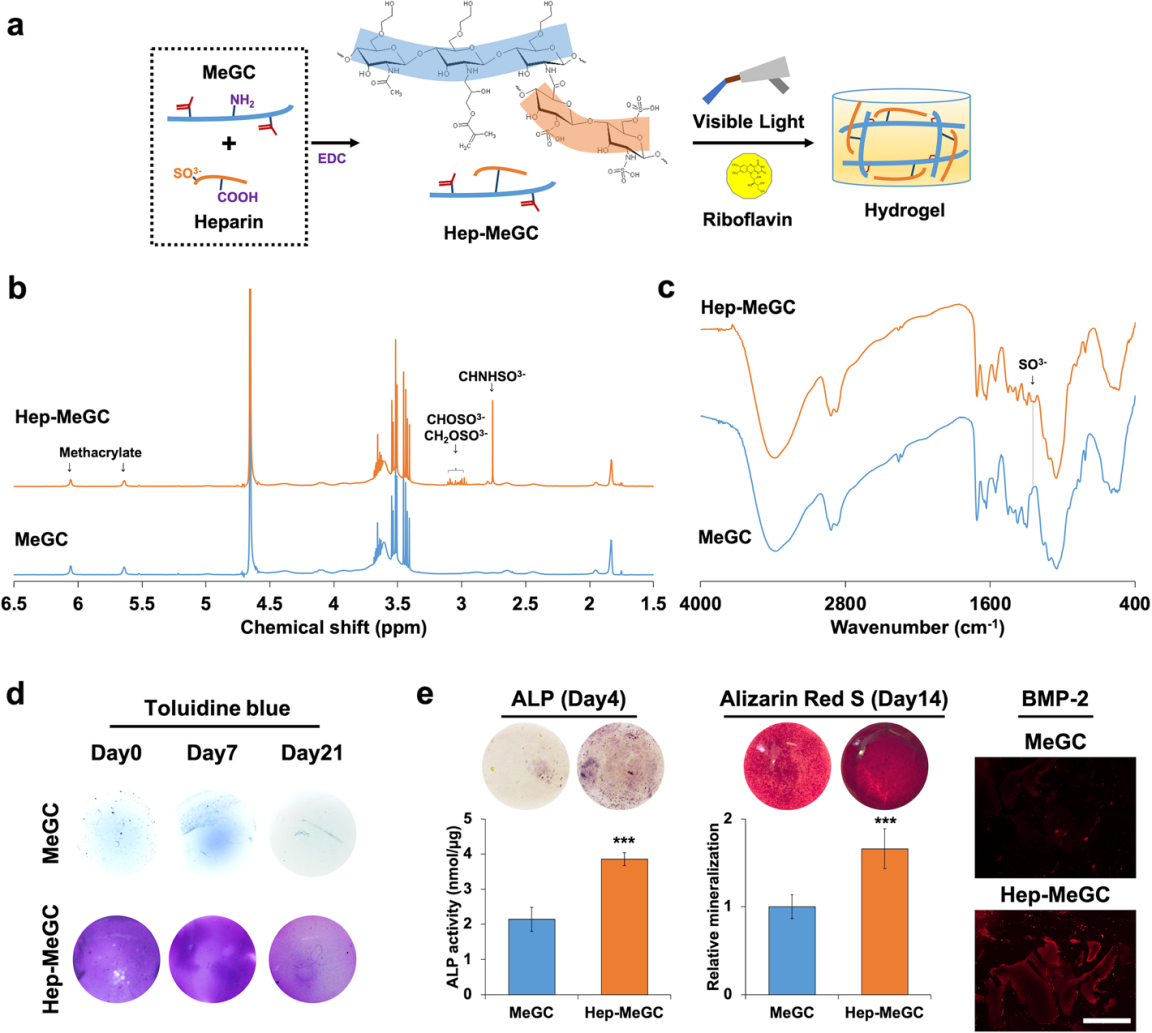
Characterization of heparinized chitosan (Hep-MeGC). **a** Chemical reaction to prepare Hep-MeGC from MeGC and heparin via EDC reaction, and visible-light induced hydrogel fabrication with riboflavin as a photoiniator. **b**^1^H NMR spectra of MeGC and Hep-MeGC (400 MHz, D_2_O) to verify heparin conjugation. **c** FTIR spectra of MeGC and Hep-MeGC to confirm heparin conjugation. **d** Toluidine blue staining images for 3 weeks to confirm stable incorporation and homogenous distribution of conjugated heparin in hydrogels. **e** Osteogenic efficacy of Hep-MeGC with encapsulated BMSCs. ALP staining and activity at day 4. Alizarin red S staining and relative mineralization at day 14. Immunostaining of BMP-2 in hydrogels encapsulated with BMSCs to demonstrate the sequestering ability of hydrogel. Scale bar is 200 μm. ***p < 0.001

Hep-MeGC was prepared by conjugation of heparin into MeGC via EDC chemistry and a hydrogel was fabricated by visible blue light crosslinking with a riboflavin initiator (Fig. 1a). The successful modification of heparin was confirmed by ^1^H NMR that showed the increment of sulfonate peaks (Fig. 1b). The FTIR spectra of Hep-MeGC exhibited the peak of SO^3−^ at 1200 cm^− 1^, confirming the successful conjugation of heparin (Fig. 1c). The stable and homogenous functionalization of heparin in a chitosan hydrogel was verified by toluidine blue that stained the negatively charged sulfonate groups (Fig. 1d). Heparinization enhanced the osteogenic efficacy of hydrogels as demonstrated by intensified staining of ALP and alizarin red S. The ability of heparinized chitosan to sequester endogenous BMP-2 secreted by the encapsulated BMSCs was further detected by immunofluorescence staining (Fig. 1e). These results suggested that the improved osteogenesis by heparin modification was potentially induced by BMP-2 sequestering of hydrogels.

### Heparinized chitosan stabilizing BMP-2 bioactivity

**Fig. 2:**
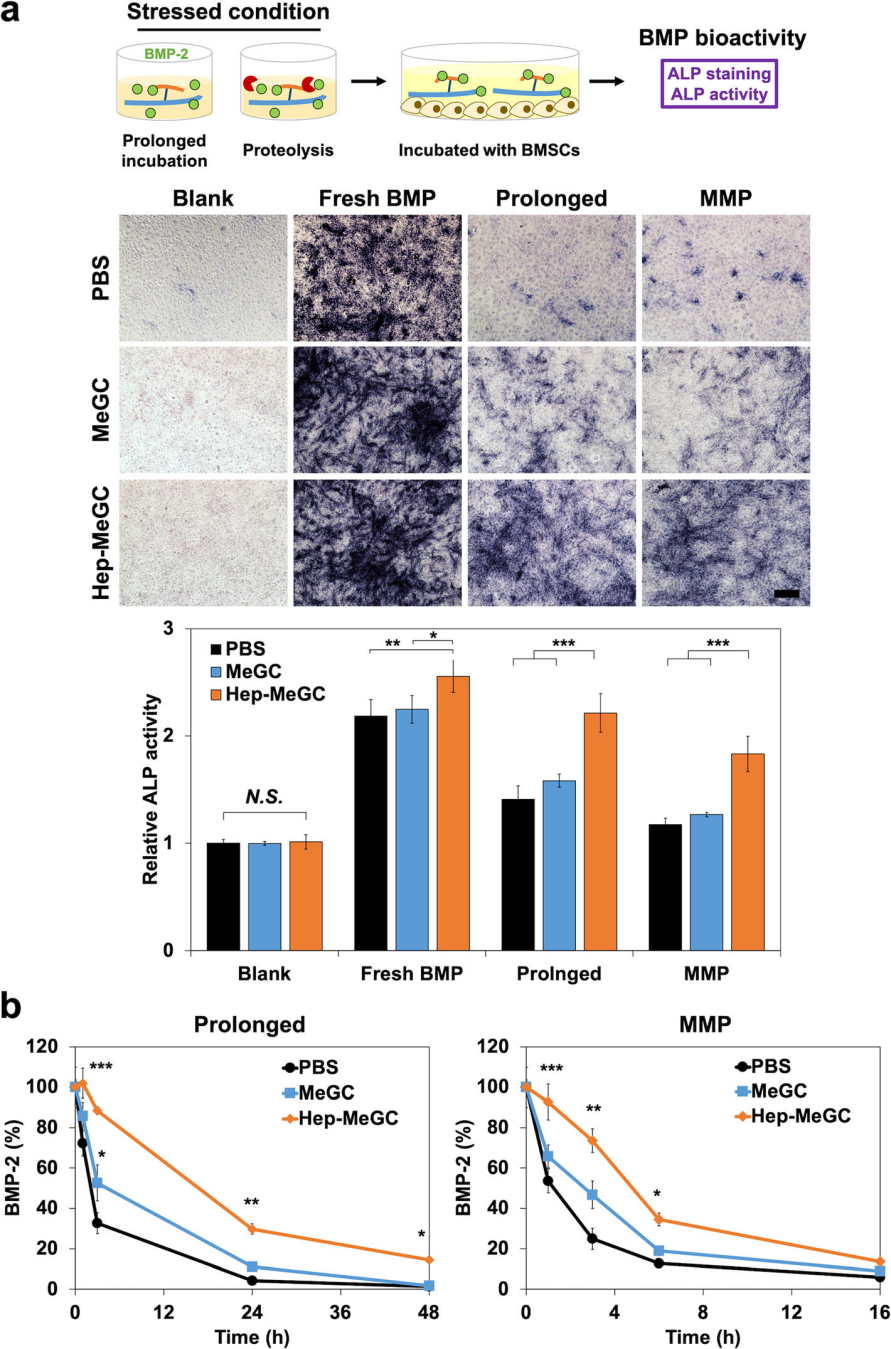
BMP-2 bioactivity test under stressed conditions. **a** The BMP-2 was incubated in prolonged condition, 37 °C for a week, and proteolysis environment, 200 ng mL^− 1^ of MMP-9 for 16 h, in the presence of Hep-MeGC. Then, it was treated with BMSCs for 4 days, and ALP staining and activity test were performed respectively to verify the bioactivity of BMP-2. *N.S.*, Not significant. *p < 0.05, **p < 0.01, and ***p < 0.001. Scale bar is 100 μm. **b** Half-life extension of BMP-2 induced by Hep-MeGC. BMSCs were cultured with 100 ng mL^− 1^ of BMP-2 with MeGC (blue) and Hep-MeGC (orange) for prolonged condition and with 200 ng mL^− 1^ MMP-9 for proteolysis condition. The concentration of BMP-2 in culture medium were quantified by BMP-2 enzyme-linked immunosorbent assay. *p < 0.05, **p < 0.01, and ***p < 0.001 compared to PBS group

The BMSCs were used to evaluate the bioactivity of BMP-2 associated with heparinized chitosan after the exposure to different stressed conditions (Fig. 2a). The BMP-2 was incubated with PBS, MeGC, or Hep-MeGC in physiological pH for 1 week at 37 °C or in MMP-9 solution for 16 h at 37 °C to mimic protease-enriched bone healing environments. The ALP staining and activity tests were performed after the treatment of with BMP-2 incubated for 4 days to verify the bioactivity of BMP-2 after exposure to the stressors. The groups without BMP-2 and with freshly prepared BMP-2 were used as a negative and a positive control, respectively. The ALP activity was gradually enhanced with the addition of MeGC and Hep-MeGC. This is possibly due to the increased half-life of BMP-2 with the addition of chitosan or heparinized chitosan in comparison to PBS group (Fig. 2b) under both prolonged and MMP conditions. The bioactivity of treated BMP-2 dropped significantly in all groups but Hep-MeGC showed the higher bioactivity compared with MeGC and PBS. Hep-MeGC demonstrated higher protective effects compared with MeGC and PBS in both prolonged and MMP-9 treated conditions.

### Heparinized chitosan protecting BMP-2 from noggin

**Fig. 3:**
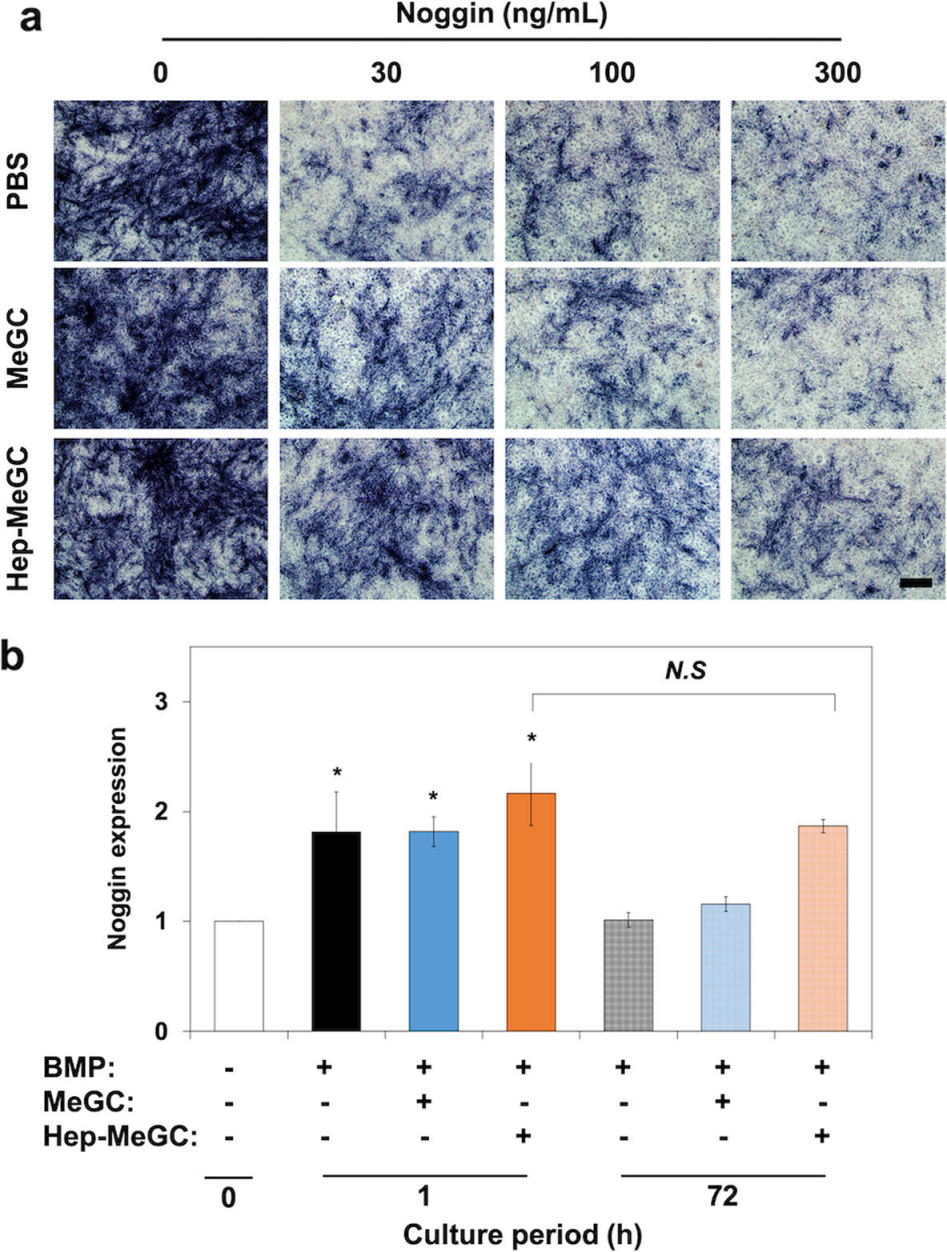
Protection effect of BMP-2 from noggin. **a** ALP staining of BMSCs cultured with graded dosages of noggin, BMP-2, and either MeGC or Hep-MeGC. Scale bar is 100 μm. **b** Noggin gene expression induced by BMP-2 in BMSCs. BMSCs were treated with BMP-2 up to 72 h. *N.S.*, Not significant. *p < 0.05 compared to the negative control (white bar)

Noggin is a BMP antagonist and inhibits BMPs signaling. The BMSCs were cultured with BMP-2 (100 ng mL^− 1^) and various concentrations of noggin (0, 30, 100, and 300 ng mL^− 1^). The ALP expression started to decrease with the addition of 30 ng mL^− 1^ noggin in PBS group (Fig. 3a and Fig. S1). The BMSCs incubated with MeGC exhibited higher ALP expression compared with PBS only group with 30 ng mL^− 1^ noggin. Furthermore, BMSCs incubated with Hep-MeGC have more intensified staining up to the addition of 100 ng mL^− 1^ noggin. Then, we investigated whether the expression level of noggin as an early response gene of BMP-2 [29] maintained with the addition of MeGC or Hep-MeGC. After 1 h of stimulation of BMP-2, noggin mRNA expressions of BMSCs were increased regardless of chitosan supplement (Fig. 3b). The level of noggin expression after 72 h decreased for BMP only and MeGC groups but did not drop for Hep-MeGC group. These results indicated that heparinized chitosan enhanced BMP signaling by protecting it from noggin.

### Characterization of hydrogel-DBM composite

**Fig. 4:**
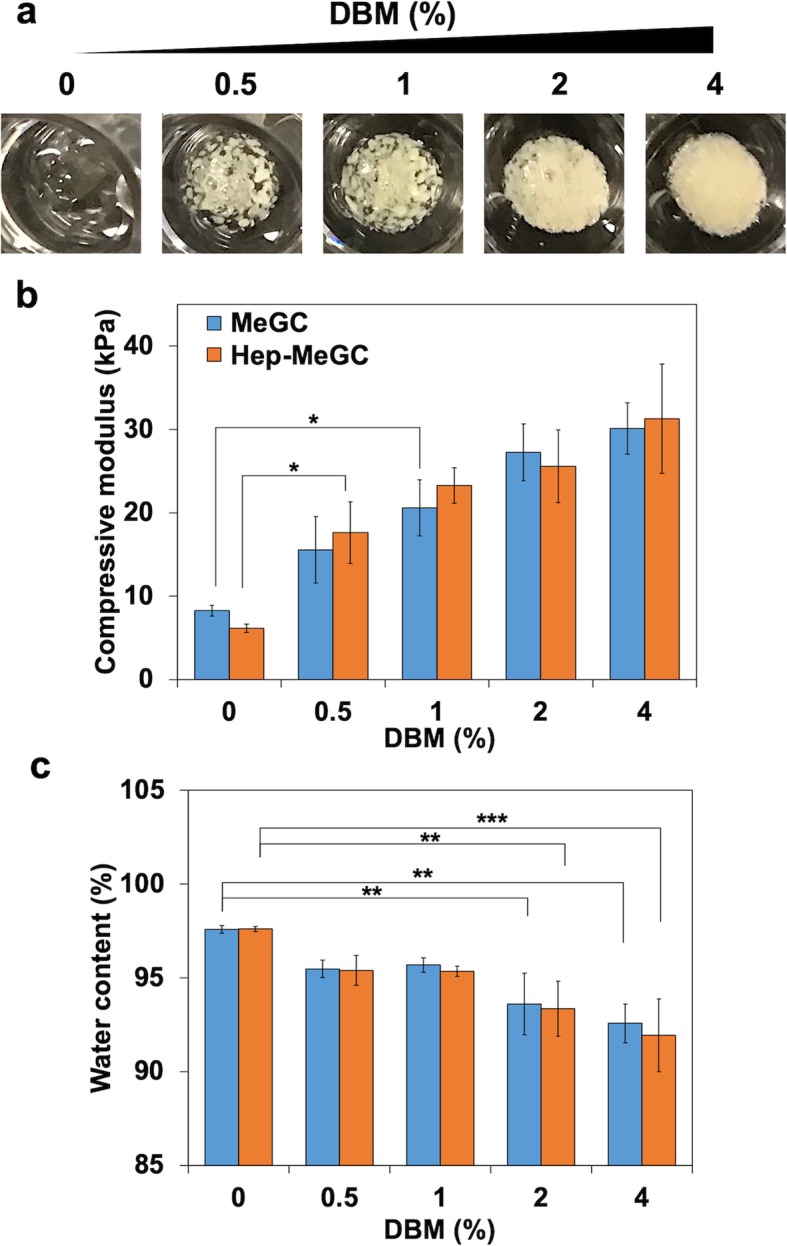
Characterization of hydrogel-DBM composites. **a** The images of composites with various concentration of DBM weight ratio, 0, 0.5, 1, 2, and 4% (w/v) in 2% hydrogel. **b** Compressive modulus of MeGC-DBM and Hep-MeGC-DBM composites. **c** Water content of composites. *p < 0.05, **p < 0.01, and ***p < 0.001

DBM was incorporated in heparinized chitosan to form osteoinductive hydrogel-DBM composites. The chitosan hydrogel was mixed with various ratio of DBM and the incorporated DBM was homogenously distributed in the hydrogel-DBM composite (Fig. 4a). Compressive modulus of the composites increased with the addition of DBM in a dose-dependent manner (Fig. 4b). Mechanical strength of the composites also increased with the addition of DBM but was not significantly different based on hydrogel types (MeGC or Hep-MeGC). Water content of the composites decreased with the addition of DBM but was not affected by hydrogel types (Fig. 4c). From these results, physical properties of the composites were not significantly affected by hydrogel types but the incorporation of DBM.

### Proliferative potential of cells in hydrogel-DBM composite

**Fig. 5:**
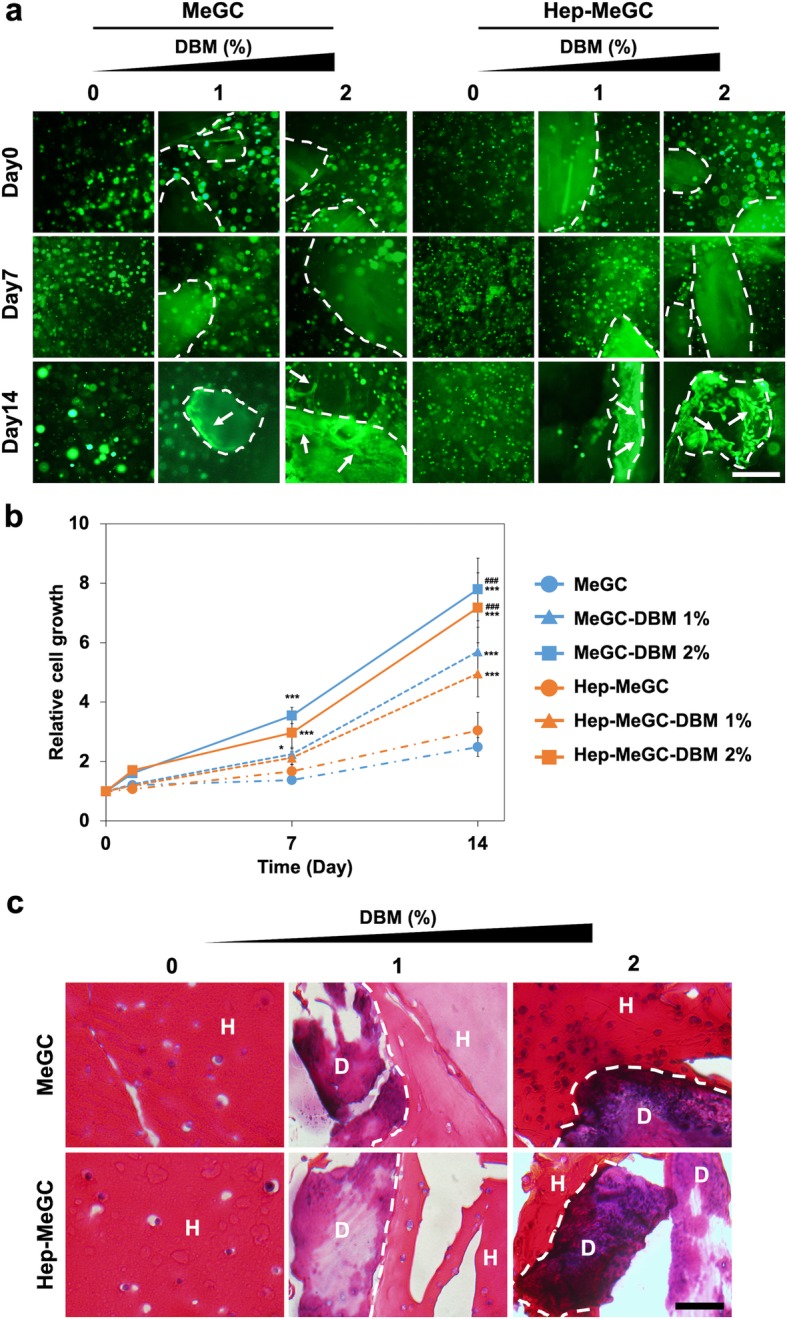
Proliferative potential of BMSCs in hydrogel-DBM composites. **a** Morphological observation of BMSCs in composites. Dotted lines demarcate the DBM/hydrogel interface and arrows point to spreading cells. Scale bar is 200 μm. **b** The proliferative potential of BMSCs measured by alamarBlue. (*) compared to MeGC or Hep-MeGC and (#) compared to MeGC-DBM 1% or Hep-MeGC-DBM 1%. *p < 0.05, ***p < 0.001, and ###*p* < 0.001. **c** H&E staining images of BMSCs cultured in composites for 2 weeks to observe the internal structure of cells and composites. Dotted lines demarcate the DBM/hydrogel interface (H: Hydrogel, D: DBM). Scale bar is 50 μm

The live/dead images of BMSCs in composites were monitored for 2 weeks. High cell viability over 90% was observed for all groups at day 0. There was no significant change in cell viability over the culture period while cell viability decreased to 85% for DBM-free groups by day 14 (Fig. S2). The morphology of cells was significantly changed with the addition of DBM which showed extended spreading alongside of DBM (Fig. 5a). The cell spreading in the composites also influenced the overall growth as confirmed by the alamarBlue result (Fig. 5b). The proliferative potential of cells increased with the incorporation of DBM in a dose dependent manner. In order to observe the interaction of DBM with the encapsulated BMSCs in composites, H&E staining was performed on the histological section (Fig. 5c). An intense purple color was observed in the DBM hydrogel composite, indicating proliferation of encapsulated cells, while cell-free constructs stained with light- or dark-pink (Fig. S3).

### Osteogenic effect of hydrogel-DBM composite

**Fig. 6:**
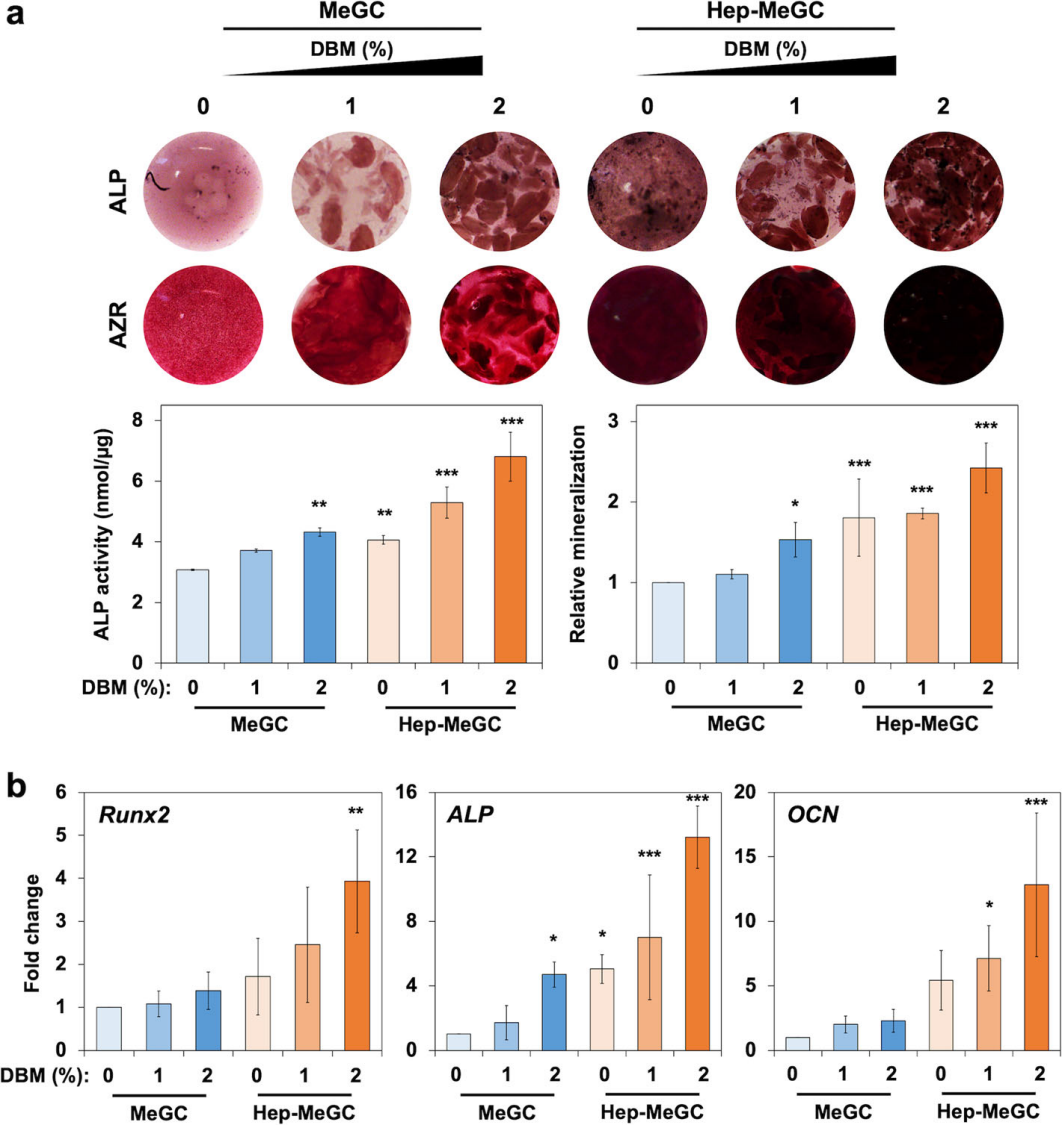
Osteogenic efficacy of hydrogel-DBM composites. **a** ALP (top) at 1 week and alizarin red S (bottom) at 3 weeks staining images of composites. ALP activity and relative mineralization were evaluated at the same day of staining. *p < 0.05, **p < 0.01, and ***p < 0.001 compared to DBM-free group (MeGC-DBM 0%). **b** Osteogenic gene expression measured by qRT-PCR at day 4 (*Runx2* and *ALP*) and day 21 (*OCN*), compared to MeGC. *p < 0.05, **p < 0.01, and ***p < 0.001 compared to DBM-free group (MeGC-DBM 0%)

The hydrogel-DBM composites provided osteoinductive environment to the encapsulated BMSCs by sequestering the cell-produced or DBM-released BMPs. The encapsulated BMSCs in composites were stained with ALP and alizarin red S at day 7 and day 21, respectively (Fig. 6a). The strengthened ALP production and activity were observed with the addition of DBM, and the trend was more escalated when heparinized chitosan was used. The mineralization result verified the augmented calcium deposition with the incorporation of DBM as well as heparin functionalization. The DBM and heparinization also upregulated the osteogenic gene expression (Fig. 6b). The *Runx2*, a transcription factor associated with osteoblast, significantly enhanced at day 4 in MeGC-DBM 2% and heparinized groups (Hep-MeGC, Hep-MeGC-DBM 1%, and Hep-MeGC-DBM 2%). The *ALP* expression was more dynamically increased with the addition of DBM as well as heparinization. The late osteogenic marker, *OCN*, also confirmed the enhanced osteogenesis with heparin and DBM incorporation.

## Discussion

Although its clear utility is reflected in its market position for skeletal reconstruction, more extensive use of DBM is hampered by its unpredictable osteoinductivity and low bone formation rate due to source- or process-related variations [30, 31]. Current DBM products often employ polymeric carriers for better handling properties but such excipients generally have no bioactive function [7]. In this work, we demonstrated that chemically functionalized chitosan with heparin (Hep-MeGC) can stabilize BMP-2 and increase its biological activity for osteogenesis. Moreover, Hep-MeGC can augment endogenous BMP activity by sequestering and localizing the cell-produced BMPs that should synergistically stimulate osteogenesis induced by DBM.

In order to investigate the underlying mechanism of heparinized chitosan to stabilize BMP bioactivity, we incubated rhBMP-2 with MeGC or Hep-MeGC under therapeutically related stressors. First of all, Hep-MeGC maintained BMP-2 bioactivity in the prolonged incubation condition, but the bioactivities in PBS and MeGC groups were reduced significantly (Fig. 2a). This was possibly due to the increased half-life of BMP-2 with Hep-MeGC which was verified by quantification of BMP-2 at both prolonged and MMP-treated conditions (Fig. 2b). BMP-2 quantification near cell layer area were also measured to verify that BMP-2 was not bound near cell layer but degraded (Fig. S4). The addition of heparinized chitosan maintained the higher concentration of BMP-2 in culture medium compared with chitosan only or nontreated groups. We also assessed the amount of BMP-2 in cell layers which exhibited less than 5% in the presence or in the absence of heparinized chitosan. This suggested the vanishing of BMP-2 was not a result of accumulation on the cell layer but a result of degradation [21]. The observed stabilizing effect of unmodified MeGC could be attributed to intermolecular interactions between MeGC and BMP-2 to form chitosan-BMP complexes, preventing protein denaturation and aggregation, thus conferring greater stability.

Many proteolytic enzymes such as MMP-2, MMP-9, or cathepsin-K were involved in bone remodeling process [32], and MMPs were key degrading enzyme expressed in either physiological [33] or pathological conditions including osteoarthritis [34], and osteoporosis [35]. The concentration of MMP-2 increased around two times, and MMP-9 increased around ten times under bone formation and disease environment [36–38]. In this MMP-9 enriched condition, we observed all PBS, MeGC, and Hep-MeGC groups suffered decrement of BMP-2 bioactivity (Fig. 2a). However, BMP-2 activity in Hep-MeGC was significantly higher than PBS and MeGC groups, indicating the most protective effects of Hep-MeGC under enzymatic stressors. MeGC only group had a minimal protective effect, suggesting interaction through heparin is essential to stabilize BMP-2 bioactivity rather than interacting with chitosan chains.

Hep-MeGC may also interact with BMP antagonists to regulate BMP functions. Noggin is a specific antagonist of BMP-2 and restricts its interaction with receptors to regulate increased levels of BMP stimuli [29]. Noggin is also known to bind S domains of heparan sulfate like BMP-2 [39, 40] and its interaction with heparan sulfate proteoglycan can form the activity gradients of BMP-2 by modulating the diffusion of noggin [20]. As such, heparin enabled BMPs to preserve longer in the culture medium indeed in the presence of noggin (Fig. 3a and Fig. S1) [21].

Lastly, we evaluated whether heparinized chitosan can enhance the osteogenic efficacy of the incorporated DBM. The compressive modulus of hydrogel-DBM composite significantly increased with the incorporation of 1% (w/v) DBM and the water content significantly decreased with the addition of 2% (w/v) DBM (Fig. 4b, c). Therefore, we selected two DBM concentration (1 and 2%) to fabricate hydrogel-DBM composite for further studies. Both ALP and mineral productions were enhanced with DBM incorporation as well as heparin functionalization (Fig. 6a). In particular, expression of *Runx2* was significantly enhanced in dual modification group (Hep-MeGC-DBM 2%), indicating that DBM synergistically enhanced osteogenesis with heparin (Fig. 6b). We also examined whether DBM-hydrogel composite can sequester BMP-2 released from DBM. BMP-2 immunostaining results showed that heparinized chitosan was able to sequester BMP-2 not only secreted from the encapsulated cells, but also released from DBM (Fig. S5).

In addition, DBM incorporation provided a cell-favorable environment in the hydrogels by increasing cell spreading and proliferation (Fig. 5). The main component of DBM mass was a type I collagen which contributed to physical and biological properties to the matrix [41]. In our previous studies, the incorporation of type I collagen or its derivatives into MeGC could enhance the spreading, proliferation, and osteogenic differentiation of the encapsulated cells [23, 42]. Herein, the observed increase in cell spreading and proliferation in the composites were possibly due to the incorporation of collagen matrix through DBM. Cell spreading become evident adjacent to DBM at 14 days of culture, while most cells remained round morphology in DBM-free hydrogels (MeGC-DBM 0% and Hep-MeGC-DBM 0%) over the culture period. Cell spreading in this study appeared to be slower and spatially non-uniform in comparison to our previous chitosan hydrogels modified with collagen or RGD peptides possibly due to the heterogeneous distribution of large DBM particles varied from 200 to 850 μm throughout the hydrogel. The spreading and proliferation of the encapsulated cells were dominantly mediated by DBM incorporation rather than heparinization.

This study proposed a novel heparinized hydrogel carrier which stabilized BMP-2 bioactivity and improved DBM osteogenic efficacy. Bone healing capacity of DBM has not been successful in clinical situations where large quantity of bone regeneration is required. We will demonstrate the efficacy of DBM hydrogel composites for bone repair in more challenging healing environments such as in large segmental defects in comparison to currently available DBM products. Additionally, future study will employ additional approaches to abrogate BMP antagonism such as utilizing an RNAi strategy to downregulate noggin expression. These strategies could maximize activity of BMP pathway in DBM-mediated osteogenesis and may increase the utility of a bone graft approach for improved bone repair. In addition, this heparinized platform could be utilized as functional polymeric carriers to deliver various proteins with heparin binding ability.

## Conclusions

Heparinized chitosan was designed by simple conjugation and photopolymerization to provide a hydrogel surface that stabilized the bioactivity of BMP-2 and potentiated the osteogenic efficacy of DBM. Heparinization preserved BMP-2 bioactivity under stressors conditions mimicking a bone healing environment and protected BMP-2 from its antagonist, noggin. The heparinized hydrogel promoted endogenous BMP activity by sequestering the cell-produced BMPs. The heparinized system was effective to deliver DBM as composite hydrogels and further enhanced osteogenic differentiation of encapsulated cells induced by DBM. This study suggests a promising strategy to increase effectiveness of BMPs and utility of current bone graft approaches for improved bone regeneration in conjunction with polymeric carrier systems.

## Materials and methods

### Materials

Glycol chitosan (~ 100 kDa) was purchased from Wako Chemical USA, Inc. (Richmond, VA). Glycidyl methacrylate, heparin sodium salt from porcine intestinal mucosa (heparin, ~ 18 kDa), and 1-ethyl-3-(3-dimethyl- aminopropyl)-carbodiimide (EDC) were supplied from Sigma Aldrich (St. Louis, MO). Recombinant human bone morphogenetic protein-2 was supplied from GenScript (Piscataway, NJ). Recombinant human matrix metallopeptidase-9 (MMP-9) was purchased from abcam (Cambridge, MA) and recombinant mouse noggin/Fc chimera (Noggin) was purchased from R&D Systems (Minneapolis, MN). Demineralized Bone Matrix (DBM; Demineralized Cortical Powder, 0.212–0.850 mm) was provided from MTF Biologics (Edison, NJ). Mouse bone marrow stromal cells (BMSCs, D1 ORL UVA, CRL-12424) was obtained from American Type Culture Collection (ATCC, Manassas, VA). All reagents were used without further purification.

### Preparation of heparinized chitosan and hydrogel-DBM composite

Visible-light inducible methacrylated glycol chitosan (MeGC) was prepared by the previously published methods [26, 43]. Heparin conjugated glycidyl methacyrlated chitosan (Hep-MeGC) was prepared by EDC activated reaction of heparin and MeGC. EDC solution was prepared as 23 mg in 1 mL distilled water and reacted with 8 mg of heparin for 30 min. Then, the reacted solution was mixed with 5 mL of 1% (w/v) MeGC in PBS for 16 h, dialyzed with 50 kDa tubes for 16 h and lyophilized. Hydrogel-DBM composites incorporated hydrogel and DBM in various weight ratio, 0, 0.5, 1, 2, and 4% (w/v), based on 2% (w/v) hydrogel concentration. All hydrogels were fabricated by 40 s irradiation of hydrogel prepolymer and 6 μM riboflavin initiator (100:0.5 volume ratio) under visible blue light (400–500 nm, 300 mW cm^− 2^) irradiation.

### Characterization of heparinized chitosan and hydrogel-DBM composite

Heparin conjugation was analyzed by ^1^H nuclear magnetic resonance spectroscopy (NMR) in D_2_O (Bruker ARX400 spectrometer). It was quantified by the integration of the peaks at 2.7–3.2 ppm (CHNHSO^3−^, CHOSO^3−^, CH_2_OSO^3−^) and compared with methacrylate peak at 5.6–6.1 ppm. The degree of heparin substitution was reported as 6.76%. Heparinized chitosan was also characterized by fourier transform infrared spectrophotometer (FTIR, Jasco 420) to verify the heparin conjugation. The incorporation of heparin over time was examined by toluidine blue (Sigma-Aldrich, MO) staining that the hydrogels were stained with 1% (w/v) toluidine blue in PBS and washed with PBS for 1 h.

Mechanical properties of composites were measured by compressive modulus and water content. The compressive modulus was evaluated by 1.6 mm diameter flat-ended indentation test via Instron Electro- Mechanical Testing Machines (Instron, Model 5564, Norwood, MA) using a Poisson’s ratio of 0.25 [26, 43]. Water content was calculated by the following equation where *W*_*wet*_ and *W*_*dry*_ referred wet and dry weight of hydrogels respectively.

$$ Water\ content\ \left(\%\right)=\frac{W_{wet}-{W}_{dry}}{W_{wet}}\times 100 $$

### Alkaline phosphatase staining and activity

Bioactivity of BMP-2 and osteogenic efficacy of MeGC, Hep-MeGC, and composites were evaluated by alkaline phosphatase (ALP) staining and activity. First of all, 100 ng mL^− 1^ of rhBMP-2 was incubated in the presence of 10 μL of MeGC or Hep-MeGC under different stressed conditions of 37 °C for 7 days or 200 ng mL^− 1^ of MMP-9 for 16 h. Then, 100 ng mL^− 1^ of incubated rhBMP-2 was treated to BMSCs in osteogenic media (OM) including high glucose Dulbecco’s Modified Eagle’s Medium, 10% fetal bovine serum, 1% antibiotic-antimycotic, 10 mM ß-glycerophosphate, 50 mg mL^− 1^ L-ascorbic acid, and 100 nM dexamethasone for 4 days. Second, 100 ng mL^− 1^ of rhBMP-2 was incubated in the presence of 10 μL of MeGC or Hep-MeGC with noggin (0, 30, 100, and 300 ng mL^− 1^) for 4 days. After all the culture, BMSCs were fixed with 10% formalin and incubated in solution containing nitro blue tetrazolium, 5-bromo-4-chloro-3-in doxylphosphate, 100 mM Tris (pH 8.5), 50 mM MgCl_2_, and 100 mM NaCl for 3 h. ALP activity was evaluated by colorimetric analysis using *p*-nitrophenol phosphate as a substrate. The value was read at 405 nm and normalized by total protein expression from BCA assay [23, 44].

### Real-time reverse transcription polymerase chain reaction (qRT-PCR)

The induced noggin expression by MeGC or Hep-MeGC and osteogenic efficacy of composites were examined by qRT-PCR. The RNAs were extracted from BMSCs by TRIzol (Invitrogen, CA) and RNeasy mini kit (Qiagen, CA), and then reverse-transcribed by SuperScript III kit (Invitrogen, CA). The cDNA products were amplified for 45 cycles with SYBR green in LightCycler 480 PCR system (Indianapolis, IN). The value was normalized with *GAPDH* expression and the primer sequences are: *GAPDH* (AGGTCGGTGTGAACGGATTTG and TGTAGACCATGTAGTTGAGGTCA), *Noggin* (GCCAGCACTATCTACACATCC and GCGTCTCGTCAGATCCTTCT), *Runx2* (CGGTCTCCTTCCAGGATGGT and GCTTCCGTCAGCGTCAACA), *ALP* (GTTGCCAAGCTGGGAAGAACAC and CCCACCCCGCTATTCCAAAC), and *OCN* (GGGAGACAACAGGGAGGAAAC and CAGGCTTCCTGCCAGTACCT). All experiments were triplicated.

### Proliferation of cells in hydrogel-DBM composite

The BMSCs were encapsulated in composites with concentration of 2 × 10^6^ cells mL^− 1^. The base hydrogels, MeGC and Hep-MeGC, were mixed with various DBM ratio, 0, 1, and 2% (w/v) in 2% (w/v) hydrogel. The BMSCs encapsulated in hydrogels were cultured in culture media for 2 weeks at 37 °C. The morphology of BMSCs were monitored by Live/Dead staining kit (Invitrogen, CA) at day 0, 7, and 14 and proliferative potential was measured by alamarBlue (Thermo Fisher Scientific, CA) assay at day 0, 1, 7, and 14. To investigate the further morphology change of cells near DBM, hydrogels at day 14 were fixed with 10% formalin for overnight, embedded in paraffin, and cut into 5 μm section. The sections were stained with hematoxylin and eosin (H&E) after deparaffinizing.

### Osteogenic differentiation of cells in hydrogel-DBM composite

The BMSCs encapsulated in composites were incubated in OM for 21 days to induce osteogenesis. The samples were collected at day 7 for ALP staining and activity test. At day 21, the mineral deposition in the samples were measured by alizarin red S staining after fixation. The hydrogels were incubated in 2% alizarin red S solution for 5 min and washed with PBS for overnight. All stained hydrogels were visualized by Olympus SZX16 stereo microscope. Calcium quantification was carried out by acetic acid extraction of alizarin red S stained hydrogels [45].

### Statistical analysis

All experiments were executed three times and represented as values with error bars which were the average and the standard deviation. One- or two-way analysis of variance with Tukey’s post hoc test was used for statistical analysis. A value of *p* < 0.05 was considered as significant.

## Supplementary information

**Additional file 1: Figure S1.** Relative ALP expression of BMSCs cultured with graded dosages of noggin, BMP-2, and either MeGC or Hep-MeGC (corresponding to Fig. 3a). *p < 0.05, ** *p* < 0.01, and ***p < 0.001.

**Additional file 2: Figure S2.** The dead staining images of hydrogel-DBM composites (corresponding to Fig. 5a). Scale bar is 200 μm. The cell viability for day 14 was quantified based on live and dead cells number using ImageJ. *N.S.*, Not significant. *p < 0.05.

**Additional file 3: Figure S3.** H&E staining images of hydrogel-DBM composites. The “H” indicates the location of hydrogel and “D” indicates the location of DBM with the proliferated cells. Scale bar is 50 μm.

**Additional file 4: Figure S4.** Half-life extension of BMP-2 induced by Hep-MeGC. BMSCs were cultured with 100 ng mL^− 1^ of BMP-2 with MeGC (blue) and Hep-MeGC (orange) for prolonged condition and with 200 ng mL^− 1^ MMP-9 for proteolysis condition. The concentration of BMP-2 near cell layer were quantified by BMP-2 enzyme-linked immunosorbent assay.

**Additional file 5: Figure S5.** BMP-2 immunostaining of hydrogel-DBM composites without or with the encapsulated cells after one-week incubation. Scale bar is 200 μm.

## Data Availability

The datasets used and/or analyzed during the current study are available from the corresponding author on reasonable request.

## Abbreviations

ALP: Alkaline phosphatase
BMP: Bone morphogenetic proteins
BMSCs: Bone marrow stromal cells
DBM: Demineralized bone batrix
EDC: 1-Ethyl-3-(3-dimethyl- aminopropyl)-carbodiimide
FTIR: Fourier transform infrared spectrophotometer
Hep-MeGC: Heparin conjugated glycidyl methacyrlated chitosan
MeGC: Methacrylated glycol chitosan
MMP-9: Matrix metallopeptidase-9
NMR: Nuclear magnetic resonance spectroscopy
OM: Osteogenic media
qRT-PCR: Real-time reverse transcription polymerase chain reaction
