# Supplementary material for: Heparinized chitosan stabilizes the bioactivity of BMP-2 and potentiates the osteogenic efficacy of demineralized bone matrix
Source: J Biol Eng. 2020 Mar 6;14:6. doi: 10.1186/s13036-020-0231-y (PMC7059291; doi:10.1186/s13036-020-0231-y)
Supplement: Supplementary file 1 — Additional file 1: Figure S1. Relative ALP expression of BMSCs cultured with graded dosages of noggin, BMP-2, and either MeGC or Hep-MeGC (corresponding to Fig. 3a). *p < 0.05, ** p < 0.01, and ***p < 0.001. [file 13036_2020_231_MOESM1_ESM.docx]

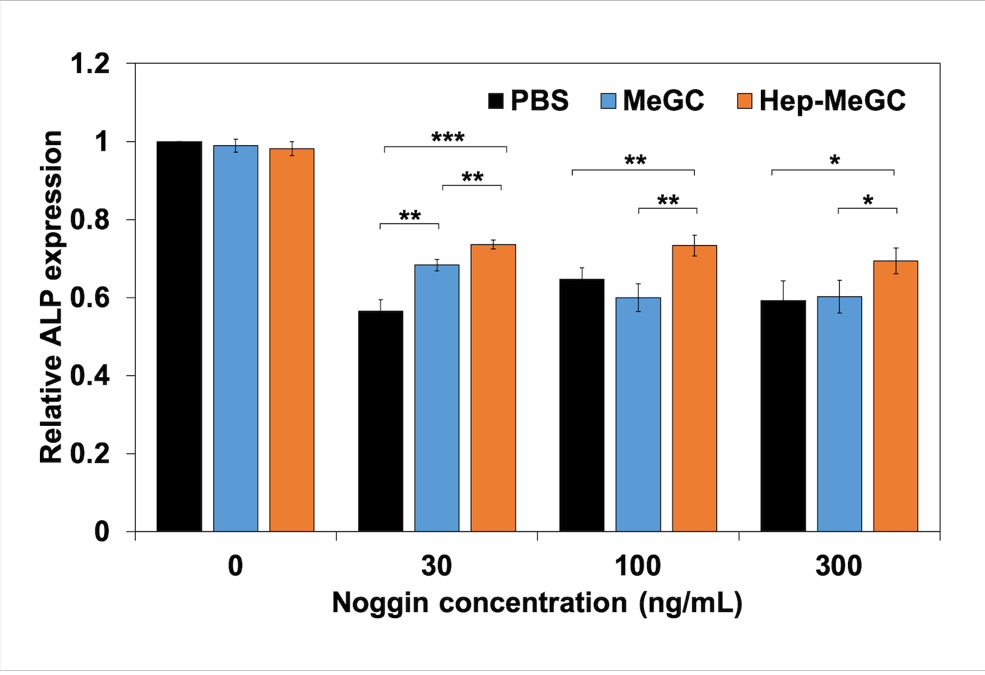


Figure S1. Relative ALP expression of BMSCs cultured with graded dosages of noggin, BMP-2, and either MeGC or Hep-MeGC (corresponding to Figure 3a). **p* < 0.05, ** *p* < 0.01, and ****p* < 0.001.
