# Supplementary material for: Heparinized chitosan stabilizes the bioactivity of BMP-2 and potentiates the osteogenic efficacy of demineralized bone matrix
Source: J Biol Eng. 2020 Mar 6;14:6. doi: 10.1186/s13036-020-0231-y (PMC7059291; doi:10.1186/s13036-020-0231-y)
Supplement: Supplementary file 2 — Additional file 2: Figure S2. The dead staining images of hydrogel-DBM composites (corresponding to Fig. 5a). Scale bar is 200 μm. The cell viability for day 14 was quantified based on live and dead cells number using ImageJ. N.S., Not significant. *p < 0.05. [file 13036_2020_231_MOESM2_ESM.docx]

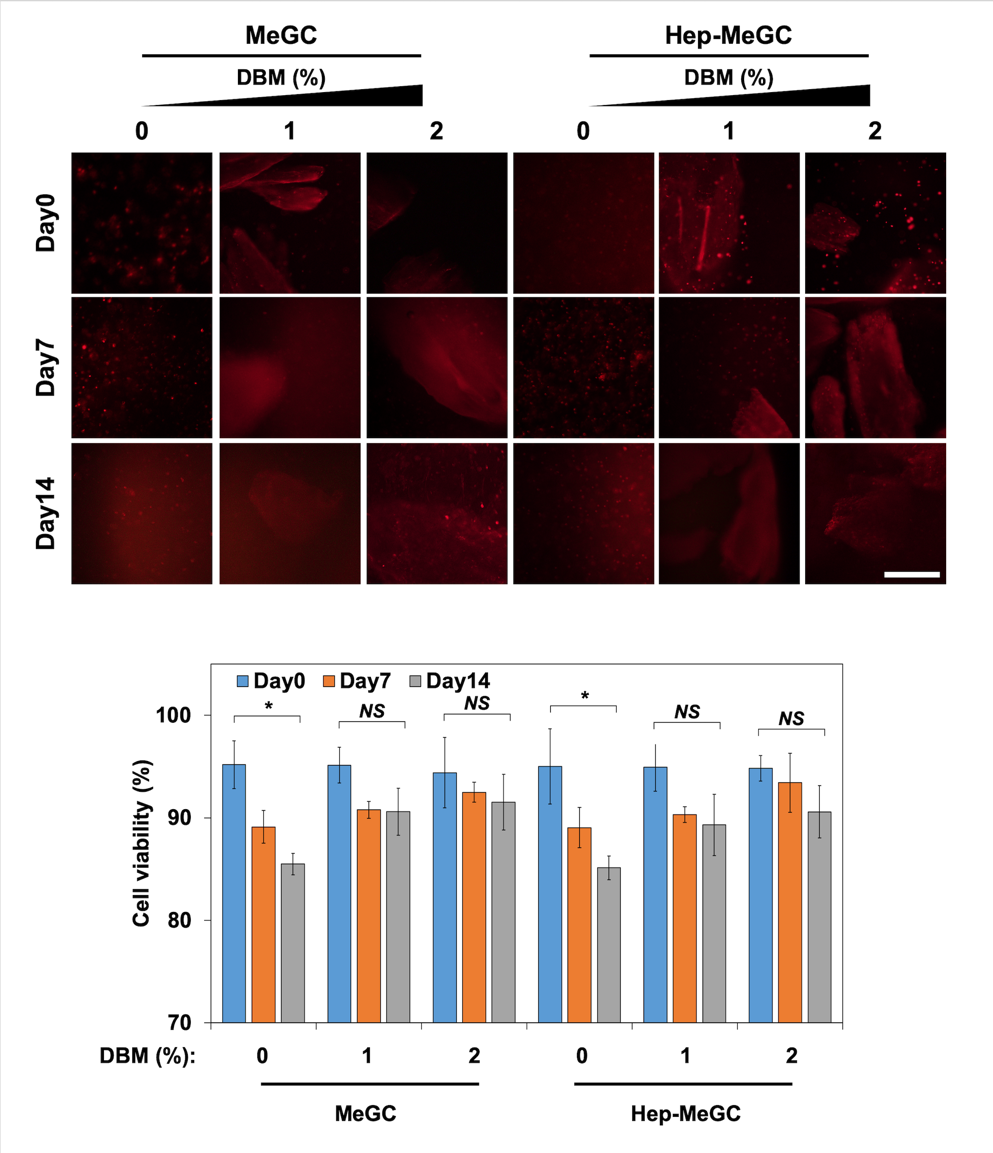


Figure S2. The dead staining images of hydrogel-DBM composites (corresponding to Figure 5a). Scale bar is 200 µm. The cell viability for day 14 was quantified based on live and dead cells number using ImageJ. *N.S.*, Not significant. **p* < 0.05.
