# Supplementary material for: Heparinized chitosan stabilizes the bioactivity of BMP-2 and potentiates the osteogenic efficacy of demineralized bone matrix
Source: J Biol Eng. 2020 Mar 6;14:6. doi: 10.1186/s13036-020-0231-y (PMC7059291; doi:10.1186/s13036-020-0231-y)
Supplement: Supplementary file 3 — Additional file 3: Figure S3. H&E staining images of hydrogel-DBM composites. The “H” indicates the location of hydrogel and “D” indicates the location of DBM with the proliferated cells. Scale bar is 50 μm. [file 13036_2020_231_MOESM3_ESM.docx]

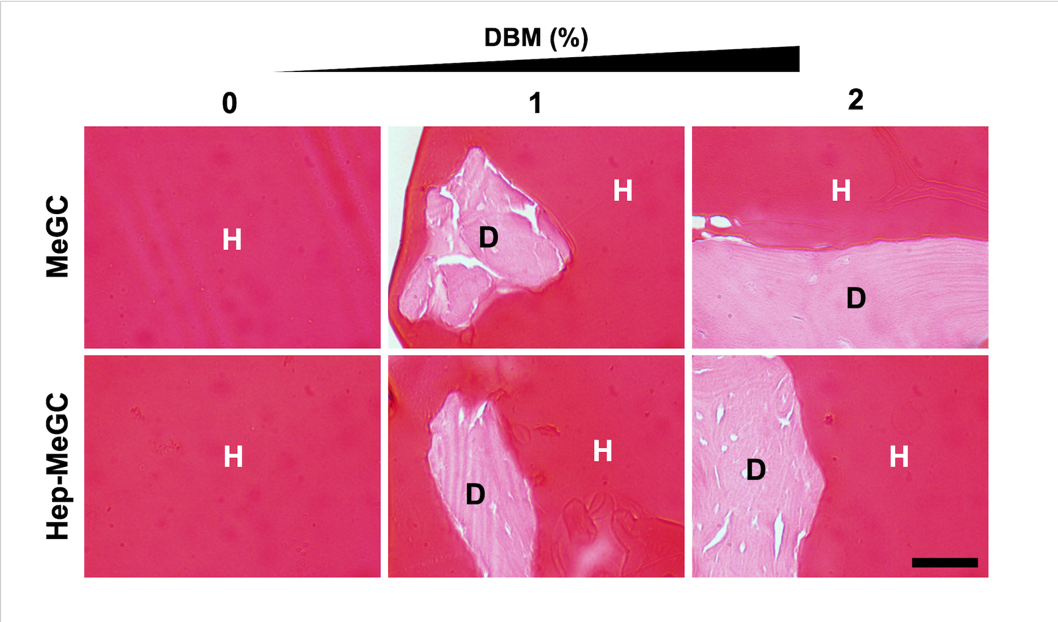


Figure S3. H&E staining images of hydrogel-DBM composites. The “H” indicates the location of hydrogel and “D” indicates the location of DBM. Scale bar is 50 µm.
