# Supplementary material for: Heparinized chitosan stabilizes the bioactivity of BMP-2 and potentiates the osteogenic efficacy of demineralized bone matrix
Source: J Biol Eng. 2020 Mar 6;14:6. doi: 10.1186/s13036-020-0231-y (PMC7059291; doi:10.1186/s13036-020-0231-y)
Supplement: Supplementary file 4 — Additional file 4: Figure S4. Half-life extension of BMP-2 induced by Hep-MeGC. BMSCs were cultured with 100 ng mL− 1 of BMP-2 with MeGC (blue) and Hep-MeGC (orange) for prolonged condition and with 200 ng mL− 1 MMP-9 for proteolysis condition. The concentration of BMP-2 near cell layer were quantified by BMP-2 enzyme-linked immunosorbent assay. [file 13036_2020_231_MOESM4_ESM.docx]

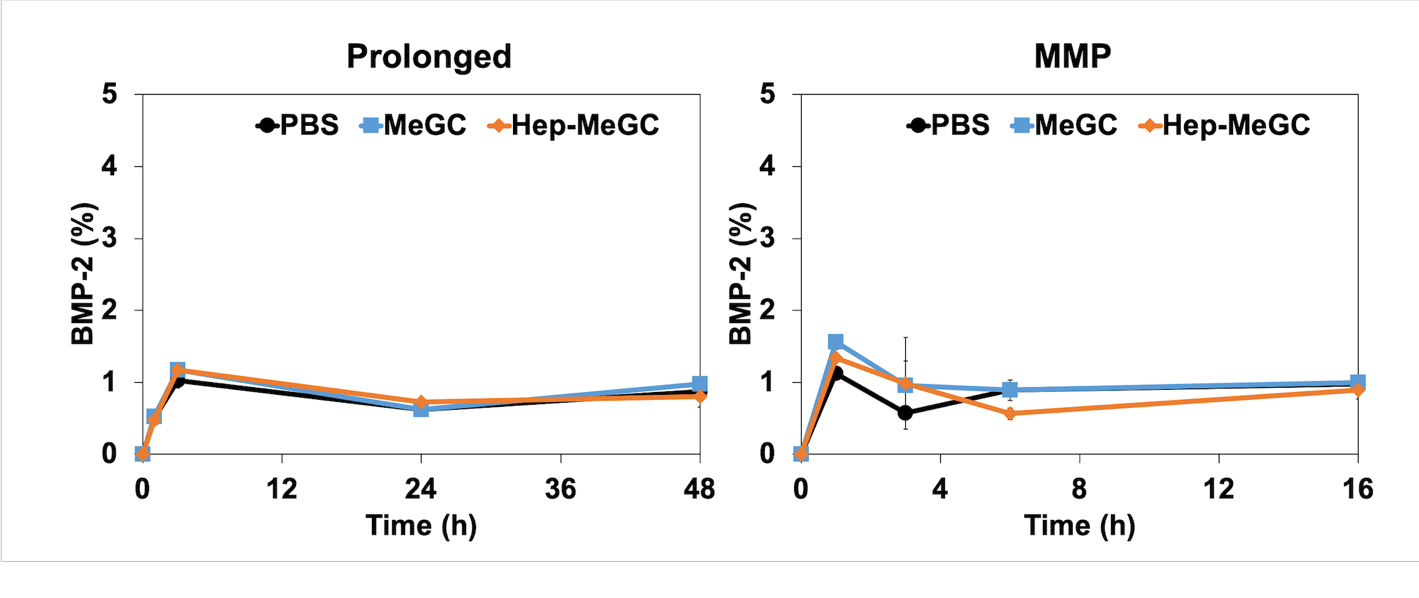


Figure S4. Half-life extension of BMP-2 induced by Hep-MeGC. BMSCs were cultured with 100 ng mL^-1^ of BMP-2 with MeGC (blue) and Hep-MeGC (orange) for prolonged condition and with 200 ng mL^-1^ MMP-9 for proteolysis condition. The concentration of BMP-2 near cell layer were quantified by BMP-2 enzyme-linked immunosorbent assay.
