# Supplementary material for: Heparinized chitosan stabilizes the bioactivity of BMP-2 and potentiates the osteogenic efficacy of demineralized bone matrix
Source: J Biol Eng. 2020 Mar 6;14:6. doi: 10.1186/s13036-020-0231-y (PMC7059291; doi:10.1186/s13036-020-0231-y)
Supplement: Supplementary file 5 — Additional file 5: Figure S5. BMP-2 immunostaining of hydrogel-DBM composites without or with the encapsulated cells after one-week incubation. Scale bar is 200 μm. [file 13036_2020_231_MOESM5_ESM.docx]

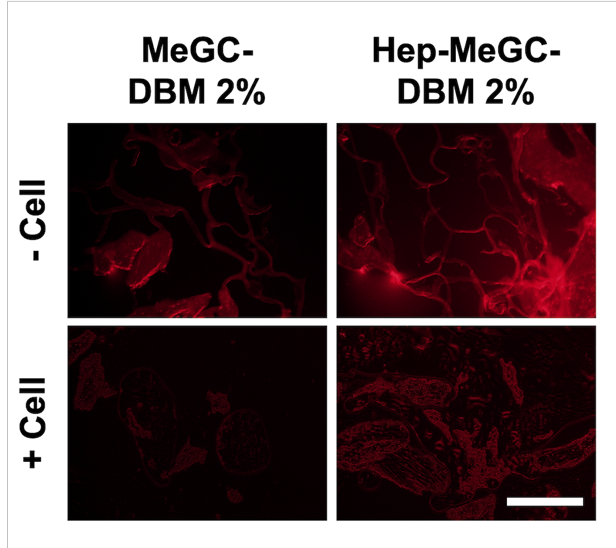


Figure S5. BMP-2 immunostaining of hydrogel-DBM composites without or with the encapsulated cells after one-week incubation. Scale bar is 200 µm.
